# Scraps

**Published:** 1895-06

**Authors:** 


### SCRAPS

PICKED UP BY THE ASSISTANT-EDITOR.

---

**Clinical Records** (11).—Doctor: "My good woman, does your son always stutter?" Mother: "Not always, sir—only when he attempts to talk."

**La façon de cueillir une femme qui se noie.**—*La Médecine moderne* fait remarquer combien sont précises les instructions données par les autorités de Dieppe aux sauveteurs chargés de surveiller les bains pendant la saison. En voici un échantillon: "Quand une dame est en danger de se noyer, ayez soin de la saisir par ses vêtements et non par sa chevelure qui pourrait parfois vous rester entre les mains."—*Gazette de Gynécologie.*

**Ambiguous.**—In an Irish daily there appeared this advertisement: "Wanted—A gentleman to undertake the sale of a patent medicine; the advertiser guarantees it will be profitable to the undertaker." This is an even unhappier mode of expression than that adopted in another paper, in which the editor said: "We regret to have to announce the death of Mr. So-and-so; but we are not astonished to hear of the sad event, as deceased had been attended for some time by Dr. S."

**To a Physician.**—Dr. J. A. Ouchterlony, in an address in the Medical Department of the University of Louisville, quoted the following lines given to him by one of his patients:

"Oh! watched for through the heavy hours
Of pain and weakness, what a gift is thine!
What a proud science, godlike and benign!
To pour on withering life sweet mercy's showers,
And on the drooping mind's exhausted powers
Like a revivifying sunbeam shine.
For thy next smile what sleepless eyelids pine!
What sinking hearts to which the summer flowers
Can breathe no joy! How many a day
I heard thy footsteps come and die away,
And clung unto that sound as if the earth,
With all its tones of melody and mirth,
To me had naught of interest, nothing worth
The brief, bright moments of thy kindly stay!"

**Sanitary Science.**—At a test-examination for sanitary inspectors, one of the candidates, when asked what a death-rate was, replied that it was a rate levied on the living to support the cemeteries. There is a weird sarcasm in this answer, and also in the reply to the question about the wilful exposure of a person suffering from an infectious disease. "He must not," said the examinee, "ride in any conveyance except a hearse without first informing the driver." Another reply to the same question laid it down as imperative that "a person dying of an infectious disease must give notice to the local authority within twenty-four hours." A candidate who evidently thought drastic measures should be employed in cases of infectious disease said that "members of a family where small-pox has broken out must be sent to a hospital and well boiled." Another mildly remarked that "among the precautions against small-pox vaccination might be looked for." In answer to some physiological questions, one examinee asserted that nitrogenous foods built up the "waist" of the body, and that "milk is the best food for children because it does not require any chewing." Another candidate gave the following elaborate and curious reply to some questions with regard to clothing: "In hot countries the perspiration which is in the skin is evaporated into steam, which goes up to form clouds, and comes down in the form of rain." Sometimes the answers are most illogically "mixed," as in the case of one which stated "that it would be necessary to get an order from the Sheriff to seize and destroy the Medical Officer of Health," and in another which affirmed that "many articles of food have to be adulterated to keep them pure."

**Medical Philology** (XIV.).—The *Catholicon* has "**Chawdepysse**: *stranguria.*" Mr. Herrtage's note is—

Cotgrave [1611] gives "*Pisse-chaude.* A burnt Pisse; also the Venerian flux; the Gonorrhœan, or contagious running." The Ortus [1500] curiously explains "*Stranguria:* as the colde pysse; *difficultas vrine quam guttatim micturiunt.*" "A recipe for the cure of *Chawdpys*, or strangury, is given in MS. Lincoln. Med. fo. 298." Halliwell. "Stranguria, otherwise called in Latine *stillicidium*, & of our old farriers (according to the French name) *chowdepis*, is when the horse is provoked to stale often, & voideth nothing but a few drops—which cometh, as the physitians say, either through the sharpness of the urine, or by some exulceration of the bladder, or else by means of some apostume in the liver or kidnies." Topsell, *Hist. of Four-footed Beasts*, ed. Rowland, 1673, p. 304. I know of no other instance of the word except in the curious O. Fr. poem "Des xxiii Manières de Vilains,' Paris, 1883, ed. Franc. Michel, p. 13, where we read—

"Si aient plenté de grume,

Plenté de frièvre et de gaunisse!

Et si aient le *chade-pisse*,

Mal ki les faiche rechaner,

Et plaie ki ne puist saner."

Jamieson [1808] gives "*Chaudpeece:* Gonorrhœa."

The *Promptorium* has "Cawepys, or chavepys, or strangury, sekenesse. *Stranguria.*"

Jamieson gives from Trevoux (*Dictionnaire*, 1752), the following definition of chaude-pisse: "Espece de maladie qu'on appelle autrement gonorrhée. Le mot de *chaude-pisse* a quelque chose d'obscene."

The *New English Dictionary* corrects the errors of the words in the *Promptorium*, which should read "cawdpys" and "chaudpys," and it gives as additional instances of the word:—"*a* 1387 *Sinon. Barthol.* (Anecd. Oxon.) 17 Diabetica passio . . . dicitur chaudepisse. *a* 1605 MONTGOMERIE *Flyting* 308 The snuff and the snoire, the chaud-peece, the chanker."

It will be seen from the quotations given above that chaudepisse, which is a good instance of the Norman-French influence on our language, originally described any form of dysuria, and that it was not till much later that it was used to describe gonorrhœa, emphasising only one of its prominent features.

**Surgical Instruments of Antiquity.**—Not the least of the attractions of the 1894 Bristol Meeting of the British Medical Association was the Museum of Antiquities brought by Messrs. Oppenheimer (see *Bristol Medico-Chirurgical Journal*, vol. xii., 1894, p. 208). It contained several early surgical instruments. In his *Leechdoms, Wortcunning, and Starcraft of Early England*, 1864, the Rev. Oswald Cockayne has many interesting observations on the subject. He says (Preface, pp. xxiii.-iv.) that in the Museo Borbonico are described some surgical instruments of bronze discovered in Herculaneum and Pompeii. There is the speculum magnum matricis, or διόπτριον, with two branches and a travelling yoke for them driven by a screw, for ocular examination of the matrix; it served rather as a dilator than as a speculum. The careful use of it is described by Paulus Ægineta (Lib. vi., cap. 73). There is also the speculum ani, or δίοπτρα, composed of two branches bent at right angles, and opening by pressure on the handles: this instrument was known as κατοπτήρ, to the author of the book on hemorrhoids among the works (sect. 6) of Hippocrates. Further has been found a forceps suited for removing pieces of bone in cases of fractured skull. It has been specially considered by Prof. Benedetto Vulpes (*Illustrazione di tutti gli Strumenti chirurgici scavati in Ercolano e in Pompei*, 1847), who thinks it may also have been intended to take up an artery. The Greeks, he observes, as appears by an inscription dug up near Athens, were able to tie an artery in order to stop hemorrhage, and words implying so much are found in a treatise of Archigenes (A.D. 100) existing in MS. in the Laurentian library at Florence: ἀποβροχθέον οὖν ἢ διαρραπτέον τὰ φέροντα τῶν ἀγγείων ἐπὶ τὴν τομήν; *the vessels carrying* (blood) *towards the incision must be tied or sewed up.* Near the end of the sixteenth century a French surgeon was the first to recover the ligature of the artery, and the instrument he used was very similar to the forceps in the Museum in Naples. A curious pair of forceps has also been found without a parallel among modern surgical instruments; the blades have a half-turn, and the grip is toothed and spoon-shaped, when closed. By construction it is suited for introduction into some internal cavity, and for holding firm and fast some excrescence there. Prof. Vulpes finds it well calculated for dealing with the excrescences which grow upon the Schneiderian membrane. or such as come on the periphery of the anus or the orifice of the female urethra; especially such as, having a large base, cannot be tied.

---

On p. 80, line 3 from bottom, for "*Herniana*" read "*Herniaria.*"

